# A Retrospective Study of Intracranial Pressure in Head-Injured Patients Undergoing Decompressive Craniectomy: A Comparison of Hypertonic Saline and Mannitol

**DOI:** 10.3389/fneur.2018.00631

**Published:** 2018-07-31

**Authors:** Feng Cheng, Min Xu, Hua Liu, Wenming Wang, Zhimin Wang

**Affiliations:** ^1^Department of Neurosurgery, Suzhou Kowloon Hospital, Soochow University, Suzhou, China; ^2^Department of Neurosurgery, The First People's Hospital of Kunshan, Jiangsu University, Suzhou, China; ^3^Department of Neurosurgery, Kunshan Hospital of Traditional Chinese Medicine, Suzhou, China

**Keywords:** traumatic brain injury, intracranial pressure, decompressive craniectomy, hypertonic saline, mannitol

## Abstract

**Objective:** The impact of hypertonic saline (HTS) on the control of increased intracranial pressure (ICP) in head-injured patients undergoing decompressive craniectomy (DC) has yet to be established. The current retrospective study was conducted to compare the effect of HTS and mannitol on lowering the ICP burden of these patients.

**Methods:** We reviewed data on patients who had sustained a traumatic brain injury (TBI) and were admitted to the First People's Hospital of Kunshan between January 1, 2012, and August 31, 2017. Patients who received only one type of hyperosmotic agent, 3% HTS or 20% mannitol, after DC were included. The daily ICP burden (h/day) and response to the hyperosmolar agent were used as primary outcome measures. The numbers of days in the intensive care unit and in the hospital, and the 2-weeks mortality rates were also compared between the groups.

**Results:** The 30 patients who received 3% HTS only and the 30 who received 20% mannitol only were identified for approximate matching and additional data analyses. The demographic characteristics of the patients in the two groups were comparable, but the daily ICP burden was significantly lower in the HTS group than in the mannitol group (0.89 ± 1.02 h/day vs. 2.11 ± 2.95 h/day, respectively; *P* = 0.038). The slope of the reduction in ICP in response to a bolus dose at baseline was higher with HTS than with mannitol (*P* = 0.001). However, the between-group difference in the 2-weeks mortality rates was not statistically significant (2 [HTS] vs. 1 [mannitol]; *P* = 0.554).

**Conclusion:** When used in equiosmolar doses, the reduction in the ICP of TBI patients achieved with 3% HTS was superior to that achieved with 20% mannitol after DC. However, this advantage did not seem to confer any additional benefit terms of short-term mortality.

## Introduction

Traumatic brain injury (TBI) is the major cause of death and severe disability among young people (1), and intracranial hypertension (ICH) accounts for about half of all deaths associated with TBI (2). Boluses of mannitol are often administered as part of the hyperosmolar treatment of ICH (3). This approach reduces intracranial pressure (ICP) and mortality rates after head injury and is superior to the use of pentobarbital for reducing the occurrence and severity of ICP elevation (4); however, its adverse effects include hypotension, electrolyte imbalance, and worsening of cerebral edema (5).

A growing body of evidence from pilot studies supports the efficacy of hypertonic saline (HTS), which has been used for hemodynamic resuscitation in shock secondary to trauma, gastrointestinal hemorrhage, burns, and sepsis (6). A number of studies have shown that decompressive craniectomy (DC) is an effective means of controlling ICH, especially in patients with traumatic lesions (7). However, very few studies have compared the efficacy of equiosmolar loads of HTS and mannitol in head-injured patients undergoing DC. Therefore, we used prospective data to compare the effect of equiosmolar doses of 3% HTS and 20% mannitol on the treatment of post-traumatic ICH in patients after DC.

## Methods

### Study population

Trained nurses collected clinical information about patients with severe TBI and entered it into the database for prospective analyses. This retrospective study was approved by the Institutional Ethics Board of the First People's Hospital of Kunshan. We reviewed data on TBI patients aged 16 years or older who were admitted to our institution between January 1, 2012, and August 31, 2017, and who underwent DC. Intraventricular ICP monitoring catheters (Codman Microsensors ICP Transducer, Codman & Shurtleff, Raynham, MA, USA) were placed during surgery. Initially elevated ICP was defined as the first recorded ICP >20 mmHg for >5 min. Additionally, patients were included if they received only one hyperosmotic agent, 3% HTS or 20% mannitol, for the treatment of intracranial hypertension. Patients were excluded if they met one of the following criteria: Glasgow Coma Scale (GCS) score of three with bilateral fixed and dilated pupils; death on Day 1; or arrival at the trauma center 24 h or more after injury. Pregnant women and patients with multiple systemic injuries were also excluded.

### Treatment protocol

All patients were managed according to the Brain Trauma Foundation guidelines (8, 9). Patients were sedated with dexmedetomidine, Propofol, or diazepam to facilitate mechanical ventilation. The aim of mechanical ventilation was to maintain SpO_2_ of >95%. Glycemic levels were targeted to around 150 mg/dl by administering insulin. The head end of the patient's bed was elevated by 15–30°. The aim of the therapy was to maintain the ICP below 20 mmHg. If, despite adequate sedation, ventilation, and head positioning, the ICP spontaneously increased to >20 mmHg for >5 min, patients received a bolus of osmotic therapy. If the osmotic agent failed to decrease the ICP to below 20 mmHg, cerebral spinal fluid (CSF) was drained until it stopped flowing spontaneously. If the ICP remained elevated, propofol or moderate hyperventilation was instituted.

### Outcome variables

The primary outcomes were daily ICP burden (h/day) and response to the hyperosmolar agent. The daily ICP burden was calculated as the mean daily duration of ICP >20 mmHg, expressed as the number of hours per day. Response to the hyperosmolar agent was documented as the ratio of the magnitude of the ICP reduction to the initial elevated ICP following the individual bolus of the hyperosmolar agent. Furthermore, we examined total number of ICU days, number of ICP monitoring days, and 2-weeks mortality. We used the recorded doses of HTS and mannitol to calculate cumulative doses.

### Statistical analyses

The goal of this study was to compare the effects of HTS and mannitol on the outcome variables described above. Descriptive summaries of the data are presented as means ± standard deviations (SDs) for continuous variables and as frequencies for categorical variables. Differences between the two groups in the baseline variables were assessed using independent-samples *t*-tests for continuous variables and chi-square tests for categorical variables. We used a *P*-value of < 0.05 for evaluating baseline differences between groups. All statistical tests were two-sided, and a *P* < 0.05 was considered statistically significant. Analyses were performed using IBM SPSS Statistics 23.

## Results

### Demographic profile

We identified 30 patients who received 3% HTS only and 30 who received 20% mannitol only for the approximate matching and additional data analyses. Table 1 shows the demographic characteristics of the patients, which were comparable between groups.

**Table 1:** Demographic characteristics.

	**HTS (*n* = 30)**	**Mannitol (*n* = 30)**	***P*-value**
Age, years	42.27 ± 17.03	41.53 ± 15.27	0.861
Female: male	6:24	5:25	0.739
Admission GCS score	5.59 ± 1.78	5.75 ± 1.32	0.563
Mechanism of head injury (*n*)			0.834
Traffic accident	17	19	
Fall	10	9	
Other	3	2	
Abnormal pupils	22	18	0.273
Predominant lesion on CT scan (*n*)			
Extradural hematoma	8	10	0.869
Subdural hematoma	12	9	
Contusion	7	8	
Diffuse injury	3	3	
Mean days of ICP monitoring	8.01 ± 2.67	8.87 ± 2.47	0.197

*HTS, hypertonic saline; GCS, glasgow coma scale; CT, computed tomography; ICP, intracranial pressure*.

### Outcome variables

The mean durations of ICU and hospital stays for the two groups were comparable (Table 2). The daily ICP burden was significantly lower in the HTS group than in the mannitol group (0.89 ± 1.02 h/day vs. 2.11 ± 2.95 h/day, respectively; *P* = 0.038). Neither the cumulative mean doses of the hyperosmolar agent (535.61 ± 74.31 ml [HTS] vs. 519.18 ± 75.54 ml [mannitol]; *P* = 0.399) nor the 2-weeks mortality rates (2 [HTS] vs. 1 [mannitol]; *P* = 0.554) of the two groups differed significantly.

**Table 2:** Study outcomes in the two groups.

	**HTS (*n* = 30)**	**Mannitol (*n* = 30)**	***P*-value**
Mean days in ICU	11.57 ± 3.65	12.37 ± 2.95	0.355
Mean days of hospital stay	33.13 ± 17.54	32.53 ± 18.27	0.897
Mean daily ICP burden (h/day)	0.89 ± 1.02	2.11 ± 2.95	0.038
Cumulative mean dose (ml)	535.61 ± 74.31	519.18 ± 75.54	0.399
2-weeks deaths (*n*)	2	1	0.554
GCS score at discharge	11.75 ± 3.11	11.90 ± 2.41	0.843

*HTS, hypertonic saline; ICU, intensive care unit; ICP, intracranial pressure*.

### ICP response

**Figure 1:**
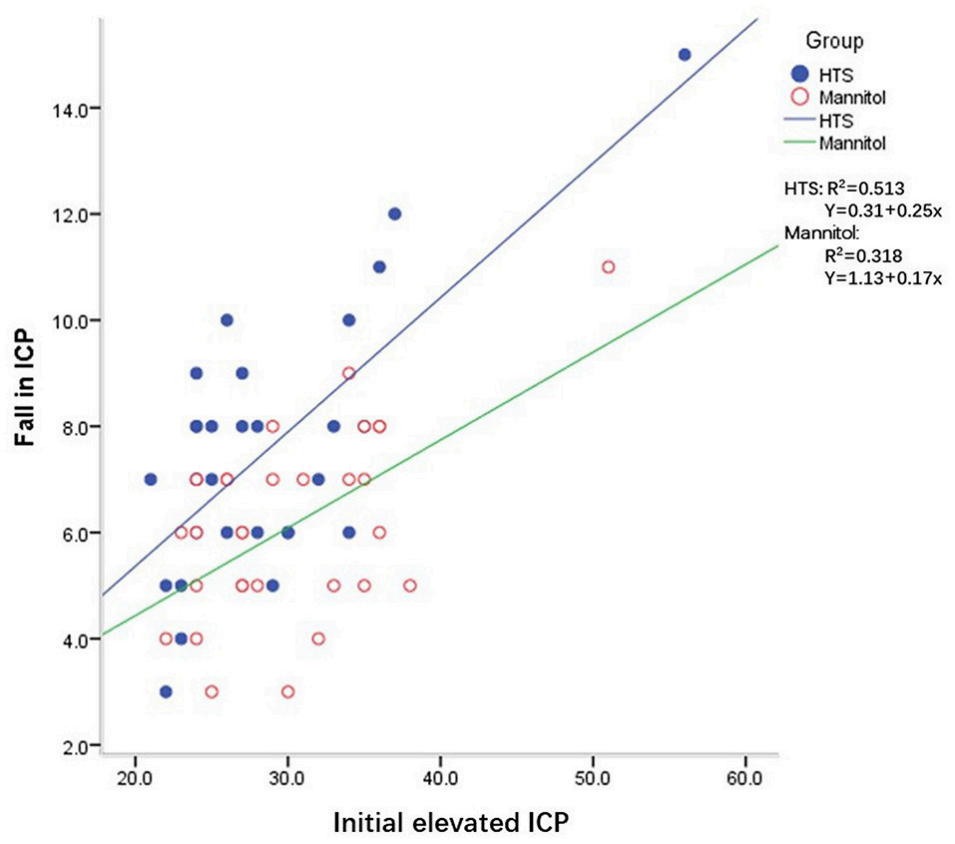
Intracranial pressure (ICP) reduction for a given ICP value with mannitol and hypertonic saline (HTS) in patients with traumatic brain injury after decompressive craniectomy.

The ICP responses to individual boluses of hyperosmolar agents are shown in Table 3. The effective doses were defined as those that returned the ICP to < 20 mmHg for >5 min after individual boluses of hyperosmolar agents were given. The number of effective doses was significantly higher in the HTS group than in the mannitol group (*P* = 0.009). When the initial ICP elevation was plotted against the reduction in the ICP after each dose of osmotic agent, the slope of the regression line was significantly steeper in the HTS than in the mannitol group (*P* = 0.001) (Figure 1).

**Table 3:** Response to individual boluses in the two groups.

	**HTS (*n* = 30)**	**Mannitol (*n* = 30)**	***P*-value**
Initial elevated ICP (mmHg)	28.43 ± 6.92	30.27 ± 6.12	0.282
ICP reduction (mmHg)	7.50 ± 2.45	6.13 ± 1.79	0.017
Efficacy of individual doses			0.009
Effective doses (*n*)	18	8	
Ineffective doses (*n*)	12	22	

*HTS, hypertonic saline; ICP, intracranial pressure*.

## Discussion

We examined the effects of HTS and mannitol on intracranial hypertension among patients with severe TBI who had undergone DC. Our findings indicated that HTS was superior to mannitol for reducing the daily ICP burden in patients with severe TBI who underwent DC. Additionally, the HTS bolus produced a more effective reduction in elevated ICP than did mannitol; however, the mortality rates of the two groups did not significantly differ.

HTS and mannitol share similar mechanisms of action for reducing elevated ICP. Both establish an osmotic gradient across the blood–brain barrier, leading to fluid shifts from the intercellular space into the microcirculation (10, 11). Indeed, several clinical studies have shown that HTS and mannitol reduce ICP and improve brain physiology to different extents. Previous studies have also demonstrated that HTS increases brain oxygenation and reduces ICP when it is used as a first-line agent and when it is used in patients with intracranial hypertension refractory to mannitol (12–14). HTS has a more pronounced and longer-lasting effect than does mannitol on elevated ICP and does not cause a rebound increase in ICP (15, 16). It causes rapid and sustained volume expansion and is effective for lowering elevated ICP when the latter is refractory to other therapies (15–18).

Three studies using equimolar doses of HTS and mannitol found that HTS was associated with either equal or greater reductions in ICP and a longer duration of effect; they also reported that mannitol produced significantly greater diuresis and volume loss (19, 20). However, conflicting results have also been reported by earlier studies that compared HTS with mannitol in patients with severe TBI (21–24). This variation appears to be related to differences in the concentrations and doses of HTS, the use of colloids in combination with HTS (21–24), and the different primary end points (6, 25).

Despite these previous studies, to our knowledge, no convincing evidence in support of the superiority of HTS over mannitol for the management of TBI patients undergoing DC has been presented. DC reduces medically refractory intracranial hypertension and is a valuable tool in the management of severe head injury (26, 27). The presence of high ICP after DC is also strongly related to unfavorable outcomes (7). We examined the daily ICP burden rather than the effects on the individual ICP spikes of repeated elevations in ICP. Sheth et al. used the “pressure time dose” (PTD) to demonstrate that the total PTD for patients with ICP > 20 mm Hg had strong predictive power for functional outcome and in-hospital mortality (28, 29). Therefore, the daily ICP burden is a meaningful outcome variable. The current study, which involved equiosmolar doses of HTS and mannitol, showed that the former had a more significant impact on the daily control of ICP after DC. Additionally, we also evaluated the responses of TBI patients who had undergone DC to an individual bolus of HTS or mannitol. The slope of the regression for a given ICP value with respect to the magnitude of the ICP reduction was higher with HTS (Figure 1). This indicates that an HTS bolus yields a more effective reduction in ICP than does mannitol when ICP is elevated, which is consistent with a previous study (30).

This study did not address the benefits of HTS on the lengths of ICU stay and hospitalization. There was also no statistically significant tendency toward a lower 2-weeks mortality rate in the HTS group. A larger cohort is required to perform the additional cost-benefit analysis between the two groups.

## Conclusion

When used in equiosmolar doses, 3% HTS is associated with a greater reduction in ICP than is 20% mannitol in TBI patients undergoing DC. A steeper ICP reduction in response to the HTS bolus was also observed. However, these advantages do not seem to confer any additional benefit terms of short-term mortality.

## Author contributions

FC and ZW designed the experiments; MX and HL performed data analysis; WW provided scientific expertize; FC wrote the manuscript.

### Conflict of interest statement

The authors declare that the research was conducted in the absence of any commercial or financial relationships that could be construed as a potential conflict of interest.
